# Significant loss of mitochondrial diversity within the last century due to extinction of peripheral populations in eastern gorillas

**DOI:** 10.1038/s41598-018-24497-7

**Published:** 2018-04-25

**Authors:** Tom van der Valk, Edson Sandoval-Castellanos, Damien Caillaud, Urbain Ngobobo, Escobar Binyinyi, Radar Nishuli, Tara Stoinski, Emmanuel Gilissen, Gontran Sonet, Patrick Semal, Daniela C. Kalthoff, Love Dalén, Katerina Guschanski

**Affiliations:** 1 Animal Ecology, Department of Ecology and Genetics, Evolutionary Biology Centre, Uppsala University, Norbyvägen 18D, 752 36 Uppsala, Sweden; 2 Institute of Animal Breeding and Husbandry, University of Kiel, Kiel, 24098 Germany; 3 Department of Anthropology, University of California, Davis, One Shields Ave, Davis, CA 95616 USA; 4 Dian Fossey Gorilla Fund International, 800 Cherokee Avenue, SE, Atlanta, GA 30315 USA; 5 Réserve de Faune à Okapis, Institut Congolais pour la Conservation de la Nature, N4, Kinshasa, Democratic Republic of the Congo; 6 Royal Museum for Central Africa, Department of African Zoology, Leuvensesteenweg 13, Tervuren, 3080 Belgium; 7 Laboratory of Histology and Neuropathology, Université Libre de Bruxelles, 1070 Brussels, Belgium; 8 Department of Anthropology, University of Arkansas, Fayetteville, AR 72701 USA; 9 Joint Experimental Molecular Unit, Royal Belgian Institute of Natural Sciences, Brussels, Vautierstraat 29, 1000 Belgium; 10 Department of Zoology, Swedish Museum of Natural History, SE-10405 Stockholm, Sweden; 11 Department of Bioinformatics and Genetics, Swedish Museum of Natural History, SE-10405 Stockholm, Sweden

## Abstract

Species and populations are disappearing at an alarming rate as a direct result of human activities. Loss of genetic diversity associated with population decline directly impacts species’ long-term survival. Therefore, preserving genetic diversity is of considerable conservation importance. However, to assist in conservation efforts, it is important to understand how genetic diversity is spatially distributed and how it changes due to anthropogenic pressures. In this study, we use historical museum and modern faecal samples of two critically endangered eastern gorilla taxa, Grauer’s (*Gorilla beringei graueri*) and mountain gorillas (*Gorilla beringei beringei*), to directly infer temporal changes in genetic diversity within the last century. Using over 100 complete mitochondrial genomes, we observe a significant decline in haplotype and nucleotide diversity in Grauer’s gorillas. By including historical samples from now extinct populations we show that this decline can be attributed to the loss of peripheral populations rather than a decrease in genetic diversity within the core range of the species. By directly quantifying genetic changes in the recent past, our study shows that human activities have severely impacted eastern gorilla genetic diversity within only four to five generations. This rapid loss calls for dedicated conservation actions, which should include preservation of the remaining peripheral populations.

## Introduction

The rate at which species and populations disappear due to human activities has dramatically accelerated over the last centuries^1–3^, leading to what has been considered the onset of the sixth mass extinction^4–6^. The major contributors to species extinctions, changes in species assemblages and decrease in population size are hunting and habitat degradation due to climate change, agricultural and urban developments, and armed conflict^3,7,8^. However, direct quantitative assessments of the impact of human activities on natural populations remain limited, causing continued discussion of the magnitude of anthropogenic effects^9^.

Recently, attention has been drawn to the dramatic rate of population extinctions and population decline^6^. Reduction in population size frequently leads to a decrease in genetic diversity^10^. Populations with low genetic diversity have limited capacities to adapt to fast changing environments^11^, display lower fertility^12^, and are prone to infectious diseases^13^. Consequently, genetic diversity is considered one of the three forms of biodiversity that deserves global conservation attention^14^. However, species with long-term low genetic diversity may have developed adaptations to mitigate the negative effects of inbreeding or have experienced genetic purging, which removes strongly deleterious variants^15,16^. In addition, genetic diversity is strongly influenced by species’ life history and demography^17,18^. Hence, simply quantifying genetic diversity is insufficient to evaluate the conservation needs of a species. Therefore, it is imperative to distinguish long-term from short-term processes and to examine temporal changes in genetic diversity. Here, museum collections play an essential role, as they frequently span the critical period of the last few hundred years during which human influence on natural ecosystems has increased. By providing a window into the past, they thus help to disentangle long-term population processes from rapid, anthropogenically driven changes, offering a unique opportunity to directly quantify human impact^19–21^.

Here we focus on two critically endangered eastern gorilla taxa, the Grauer’s (*Gorilla beringei graueri*) and mountain gorillas (*Gorilla beringei beringei*). All four recognized gorilla taxa have experienced a continued long-term population decline over the last 100,000 years. However, this decline was particularly pronounced in the eastern species^22^, which is reflected in low estimates of genetic diversity in eastern compared to western gorillas^23^. Furthermore, the two eastern subspecies differ from each other in current population sizes^24,25^. Field-based studies have estimated up to 90% population decline in Grauer’s gorillas in the last two decades due to habitat loss and poaching, with reported current population size of less than 4,000 individuals^26^. The mountain gorilla population of the Virunga Volcanoes Massif counted only 250 individuals in the early 1980s^27^, but has recovered to around 480 individuals today^24,28^ thanks to major international conservation effort. Therefore, eastern gorillas represent an intriguing case of potentially rapid population decline over the last few generations that happened against the backdrop of slow long-term reduction in population size.

Demographic analyses of gorillas have so far only considered modern samples and therefore have limited power to resolve how genetic diversity has changed in recent times^29^. We aim to fill this gap by using historical museum and modern faecal samples that span the last 110 years, thus providing a direct past-to-present comparison. To quantify anthropogenic effects on genetic diversity (and hence evolutionary potential) of eastern gorillas, we focus on complete mitochondrial genomes, a powerful and widely used marker in conservation genetics that is well suited to reflect recent demographic changes^30–32^. Due to the low effective population size of mtDNA (1/4^th^ of the nuclear genome), nucleotide variants rapidly become fixed through genetic drift, allowing accurate resolution of demographic processes, even when the sampling period encompasses only a few generations^33–35^. Changes in genetic diversity can rapidly be detected as emergence or loss of unique genetic variants for this maternally inherited, non-recombining locus, whereas longer time periods (more generations) are needed before these processes are reflected in the nuclear genome^36–38^.

The aims of our study are two-fold. First, we want to quantitatively assess how genetic diversity has changed over the course of only a few generations in two critically endangered gorilla subspecies that differ in their historical population sizes. Second, we aim to evaluate the relative contribution of decline in population size versus local population extinction to the genetic diversity of Grauer’s gorillas.

## Methods

An extensive description of methods for sample collection, extraction, hybridisation capture of complete mitochondrial genomes and sequencing procedures is presented in^39^. Briefly, 69 historical Grauer’s gorilla samples (median collection year = 1950 [1910–1980]), 64 modern Grauer’s gorilla fecal samples (collected in 2014), and 22 historical mountain gorilla samples (median collection year = 1921 [1913–1956]) were included in this study (Table S1). Historical Grauer’s and mountain gorilla samples were obtained from mainly adult individuals (age class estimates were based on skull morphology, Table S1). Therefore their expected birth date predates the time when major anthropogenic factors started to affect these subspecies^26,27^. Fecal samples were collected in 2014 in two locations in the Democratic Republic of Congo (DRC) from two social groups: one social group from the Nkuba Research and Conservation Area (NK) in the Walikale territory, and one from the high altitude sector of the Kahuzi-Biega National Park (KBNP)^40^ (Table S1). Approximate individual age class was determined by dung bolus size^41^ and we excluded juvenile samples with dung diameter <5.5 cm from further analyses (N = 8) to reduce confounding effects of having pre-dispersed maternally related individuals that share the same mitochondrial haplotype.

Double-barcoded, double-indexed sequencing libraries were generated from 40 historical Grauer’s gorilla samples, all 22 mountain gorilla samples, and three extraction blanks following^42^. The libraries were subjected to 125 bp paired-end shotgun sequencing on the Illumina Hiseq2500 platform (High Output Mode). In addition, we captured complete mitochondrial genomes from all adult fecal samples (N = 56) and historical Grauer’s gorilla samples that were not shotgun-sequenced or for which we obtained insufficient mitochondrial genome coverage after shotgun sequencing (N = 59)^39^.

### Sequence data processing

Sequences were demultiplexed, merged, mapped to the gorilla reference genome (GorGor3.1), and quality filtered as described in^39^. Complete mitochondrial sequences were obtained requiring at least 3x coverage for each nucleotide position^39^. We removed the mitochondrial D-loop before further analysis, as this hypervariable region is problematic to assemble in great apes, especially from degraded samples^43,44^, and frequently represents a major source of assembly error from shotgun data^45^. After combining data from identical individuals represented by multiple fecal samples (as determined by their multilocus microsatellite genotypes, see^40^), and removing genomes with more than 25% of missing sites, the newly generated dataset contained a total of 115 mitochondrial genomes: 68 historical Grauer’s individuals, 29 unique modern Grauer’s individuals, and 18 historical mountain gorilla individuals sequenced at an average depth of 120x (Table S1, Fig. S1).

### Sequence reliability

Sequence data from the historical samples showed low rates of typical ancient DNA damage patterns (see^39^), as is expected for archived museum samples^46,47^. As fecal samples were sonicated prior to library preparation and sequencing, no DNA damage analysis was performed for them^39^. Human contamination in all samples was low (0.94% ± 0.04, 0–2.4%) and mtDNA sequences of all replicate samples (N = 48) were identical, demonstrating the reliability of the used method (see^39^). For all polymorphic sites in Grauer’s (N = 37) and mountain gorillas (N = 4) we manually confirmed that no stop-codons were introduced by these variants. In Grauer’s gorillas we identified one polymorphic site within a non-coding region, five polymorphic sites within rRNA and tRNA genes, with the remaining 31 variants residing within coding regions. Most of the identified polymorphisms in the coding regions were either synonymous (13 out of 31) or coded for an amino acid change commonly observed in nature (15 out of 31, based on the PAM250 matrix, score ≥0). Transition accounted for the majority of substitutions (33 out of 37). In mountain gorillas, all polymorphic sites were transition within coding regions encoding for an amino acid change commonly observed in nature (PAM250 matrix ≥0).

### Mitochondrial genome analyses

We combined our data with published Grauer’s (N = 7) and mountain (N = 8) gorilla mitochondrial genomes^22,44^ (Table S2). Additionally, we included published mitochondrial genomes from western lowland gorillas (*Gorilla gorilla gorilla*, N = 43), a subspecies of the western gorillas with a large population size of ~140.000 individuals^48^ (Table S2). This was done to allow cross-species comparison of genetic diversity. Birth year of individuals from published samples ranged from after 1990 to 2006 (Table S2). Geographic coordinates of the sampling locations were obtained from the museum labels (historical samples), field records (fecal samples), or derived from publications (published sequences). All mitochondrial genomes were aligned with Clustal omega 1.2.4^49^. Minimum spanning haplotype networks were constructed in PopART V1.7 (http://popart.otago.ac.nz). We grouped all mitochondrial genomes by sub-species and sample type (historical or modern sample) and calculated haplotype statistics, molecular diversity indices, and neutrality tests in Arlequin3.5^50^. We assessed the effect of unequal sample size by using permutation tests. To this end, we randomly subsampled mitochondrial genomes from each population to match the population with the smallest sample size (modern mountain gorillas, n = 8) and repeatedly (n = 1000) recalculated haplotype and nucleotide diversity.

To accurately model eastern gorilla demography, we estimated the gorilla specific mitochondrial mutation rate using BEAST 2.4.6^51^. We used the estimated split date of western and eastern gorillas of ~1 million years ago obtained from nuclear data as calibration point^52,53^. To exclude confounding effects of different sample ages, only modern mitochondrial genomes were used. We first estimated genome wide mutation rate using the HKY-site model and enforcing a strict molecular clock. Birth-rate and clock-rate priors were set as gamma distribution with α = 0.001 and β = 1000. The prior for the split time between western and eastern gorillas was set as a log-normal distribution with M = 0.5 and S = 0.1, which corresponds to a divergence time of 0.848–1.18 Mya (95% CI). The Bayesian model was run for an MCMC length of 500 million and we used Tracer 1.6^54^ to confirm run convergence and obtain probability distributions. We then partitioned the aligned mitochondrial genomes into coding genes (splitting triplets into 1^st^ + 2^nd^ and 3^rd^ position), rRNAs, tRNAs, and non-coding regions in Geneious 10.1.2 using the annotated gorilla reference. The same BEAST model was then used to estimate partition-specific mutation rates. The obtained mutation rates were highly similar to those published for the human mitochondrial genome^55,56^ (Fig. S2, Table S3).

We used the Bayesian skyline model in BEAST 2.4.6^51^ to infer demographic changes within the last centuries in Grauer’s and mountain gorillas. The best partition scheme and the best fitting model for each partition were identified with PartitionFinder 2^57^, only considering models available in the BEAST 2 software package. The skyline analysis was then run for both subspecies separately under a strict molecular clock model using the inferred gorilla mutation rates for the different partitions and MCMC length of 500 million.

To account for the possibility of more recent demographic changes that could be bypassed by the skyline model, we also employed an approximate Bayesian computation approach^58^. Since initial exploratory analyses failed to detect the onset of demographic change within the last 2000 years due to the lack of power over this large time period, we narrowed down the time period to the last 110 years. This time frame reflects the temporal distribution of our historical samples and encompasses the documented population size decline within the last four decades in Grauer’s and mountain gorillas^26,27^. We divided our dataset into two temporal groups: present (modern samples) and historical (museum specimens).

We performed two inferences: (1) a model choice analysis with three competing models in which effective population size was constant, decreased or increased in a single recent event (30–110 years ago); and (2) a parameter estimation of two effective population sizes—present and historical. We ran 30 million simulations (10 million per model) for the model choice analysis and employed 10 acceptance proportions (0.005–0.05%) for assessing consistency of the estimated model likelihoods. For the parameter estimation analysis, we ran 1 billion simulations and applied rejection with a linear regression adjustment over the 0.001% (=10,000) accepted simulations. In a dedicated analysis, we selected the following summary statistics: number of haplotypes, number of private haplotypes and genetic diversity for each of the two temporal groups as well as pairwise differences and F_ST_ between the two temporal groups.

Finally, to discriminate between negative results that were due to lack of demographic change versus those due to limited information content of the data, we employed pseudo-observed datasets (PODs) to assess the statistical power of our samples and methodology. All analyses were performed separately for Grauer’s and Mountain gorilla mitochondrial genomes in the software BaySICS v1.9^59^ using the estimated gorilla mutation rate (1.28 × 10^−8^), no gamma parameter, and a Ts/Tv bias of 0.75 and 0.933 in mountain and Grauer’s gorillas, respectively (as identified by the selection model of molecular evolution carried out in MEGA 7^60^).

### Data accessibility

Sequence reads are available for download at the European Nucleotide Archive (ENA) under accession number PRJEB21370.

Mitochondrial genome sequences are available at Genbank under accession numbers MH177628 - MH177754.

Used python scripts are available upon request.

## Results

### Haplotype network

**Figure 1:**
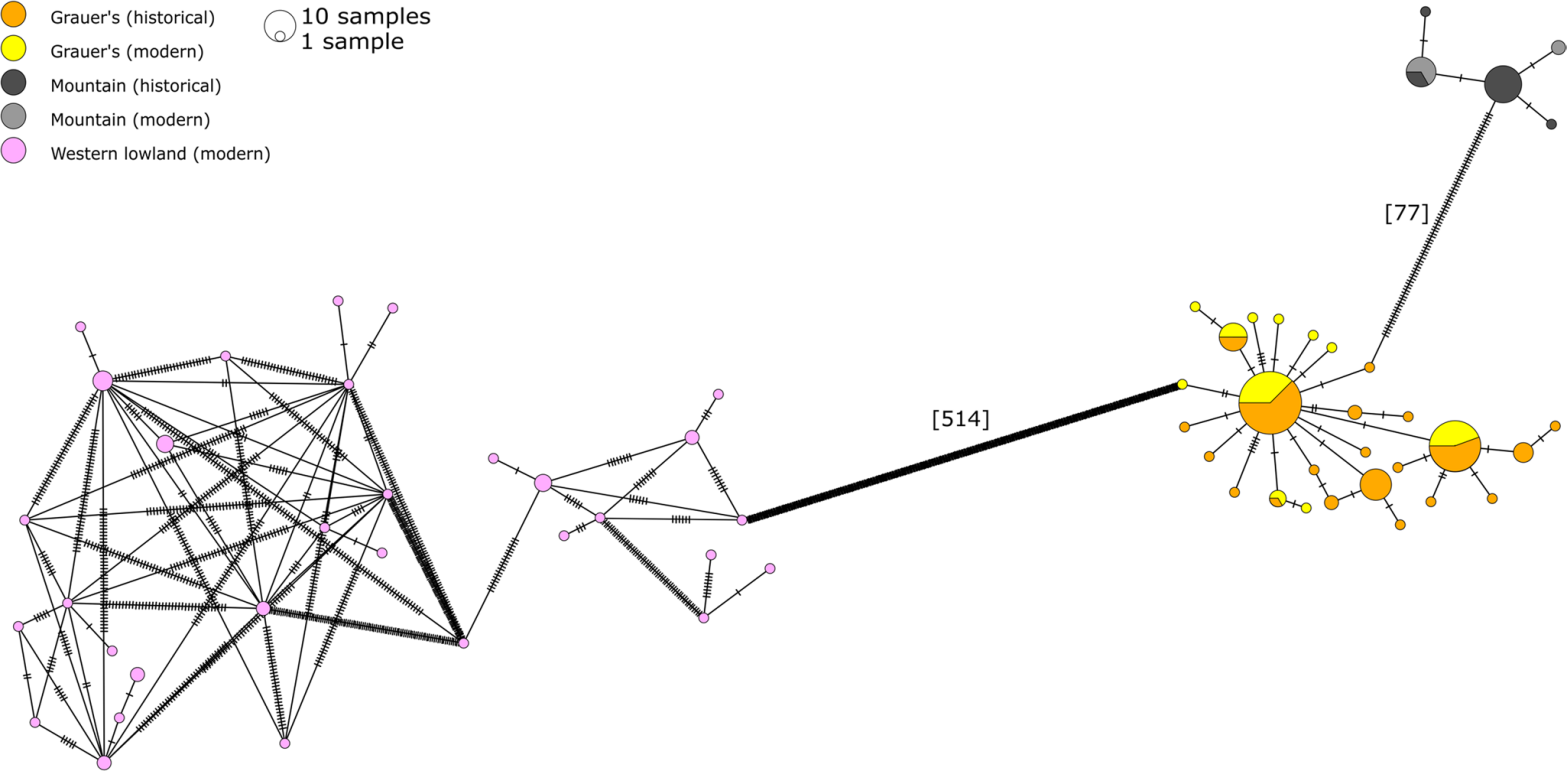
Minimum spanning haplotype network based on complete mitochondrial genomes. Haplotypes are coloured by (sub)species and sample age (historical or modern). Ticks on the haplotype edges correspond to the number of substitution. The total number of substitutions between subspecies is shown in square brackets.

The minimum spanning haplotype network clearly differentiated the three studied gorilla subspecies into separate clusters (Fig. 1). Based on the complete mitochondrial genomes (without the D-loop), we detected 32 haplotypes in western lowland gorillas, with on average 66.4 nucleotides differences between them (Fig. 1). Combining historical and modern eastern gorilla samples, we found 27 Grauer’s and five mountain gorilla haplotypes and significantly lower average number of nucleotide difference between haplotypes than in western lowland gorillas (1.6 for Grauer’s and 0.775 for mountain gorillas, P_western-Grauer’s_ < 0.001, P_western-mountain_ < 0.001). Consequently, haplotype diversity and nucleotide diversity for both eastern gorilla species was significantly lower than in western lowland gorillas (Table S4). To evaluate the effect of using only parts of the complete mitochondrial genome, we sub-sampled the genomes to cytochrome c oxidase (subunit 1–3), cytochrome b, and 16 S rRNA, regions commonly used as markers in populations genetics and phylogenetics. To this end, the target genes were extracted from the alignment based on the annotated reference (gorGor3.1) using Geneious 10.1.2^61^. After subsampling, most of the unique haplotypes could not be identified (Fig. S3), showing the importance of obtaining complete mitochondrial genomes when studying species with extremely low diversity.

### Temporal changes in genetic diversity

**Figure 2:**
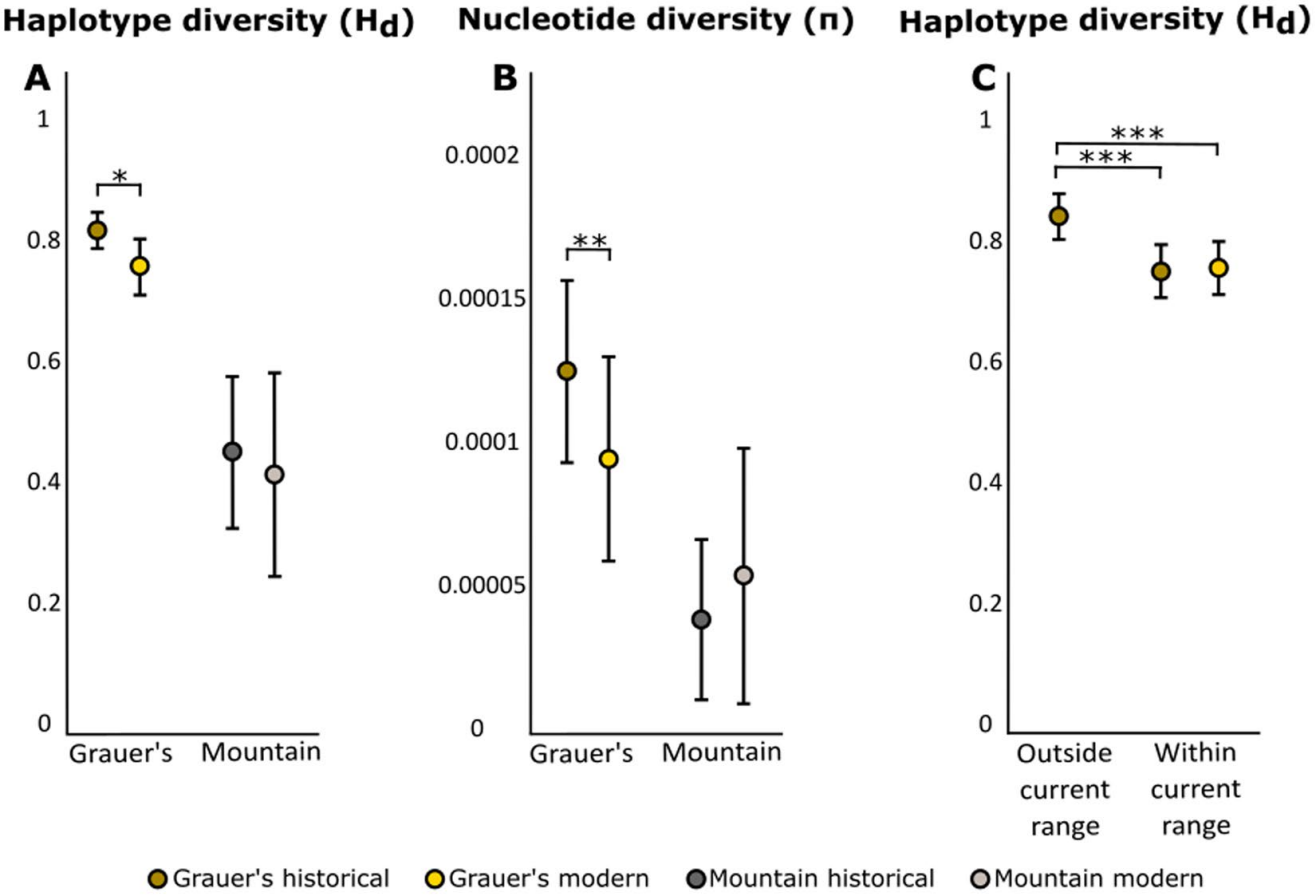
(**A**) Haplotype and (**B**) nucleotide diversity in both eastern gorilla subspecies. (**C**) Haplotype diversity in Grauer’s gorilla, grouped by geographic region and sampling period. Error bars depict 95% CI.

In Grauer’s gorillas, we identified 20 and 11 haplotypes among historical and modern samples, respectively, and a significant decline in haplotype and nucleotide diversity between the two age categories (P_Hd_ = 0.0169, P_π_ = 0.0062, 2-sample t-test, Fig. 2A,B, Table S4), which could not be explained by differences in sample size (Fig. S4, Table S5).

In mountain gorillas, we found that genetic diversity was already extremely low prior to the 1980s, with only four unique haplotypes found in historical samples that predate the reported population low. However, we found only two haplotypes in the modern samples (median birth year 1997 [1982–2012]), one that was shared with the historical samples and one unique to the modern samples. We detected no significant difference in nucleotide and haplotype diversity between modern and historical samples (P_π_ = 0.60, P_Hd_ = 0.47, 2-sample T-test), a result that is independent of sample size (Fig. S4), although the haplotype diversity mean was lower in the modern data compared to the historical data (Fig. 2A, Table S4). Historical and modern mountain gorillas were genetically less diverse than historical and modern Grauer’s gorillas, respectively (P_historial-Hd_ < 0.001, P_modern-Hd_ < 0.001, P_historial-π_ < 0.001, P_modern-π_ = 0.0046, 2-sample-T-test, Fig. 2A,B, Table S4).

### Geographic distribution of genetic diversity within Grauer’s gorilla

**Figure 3:**
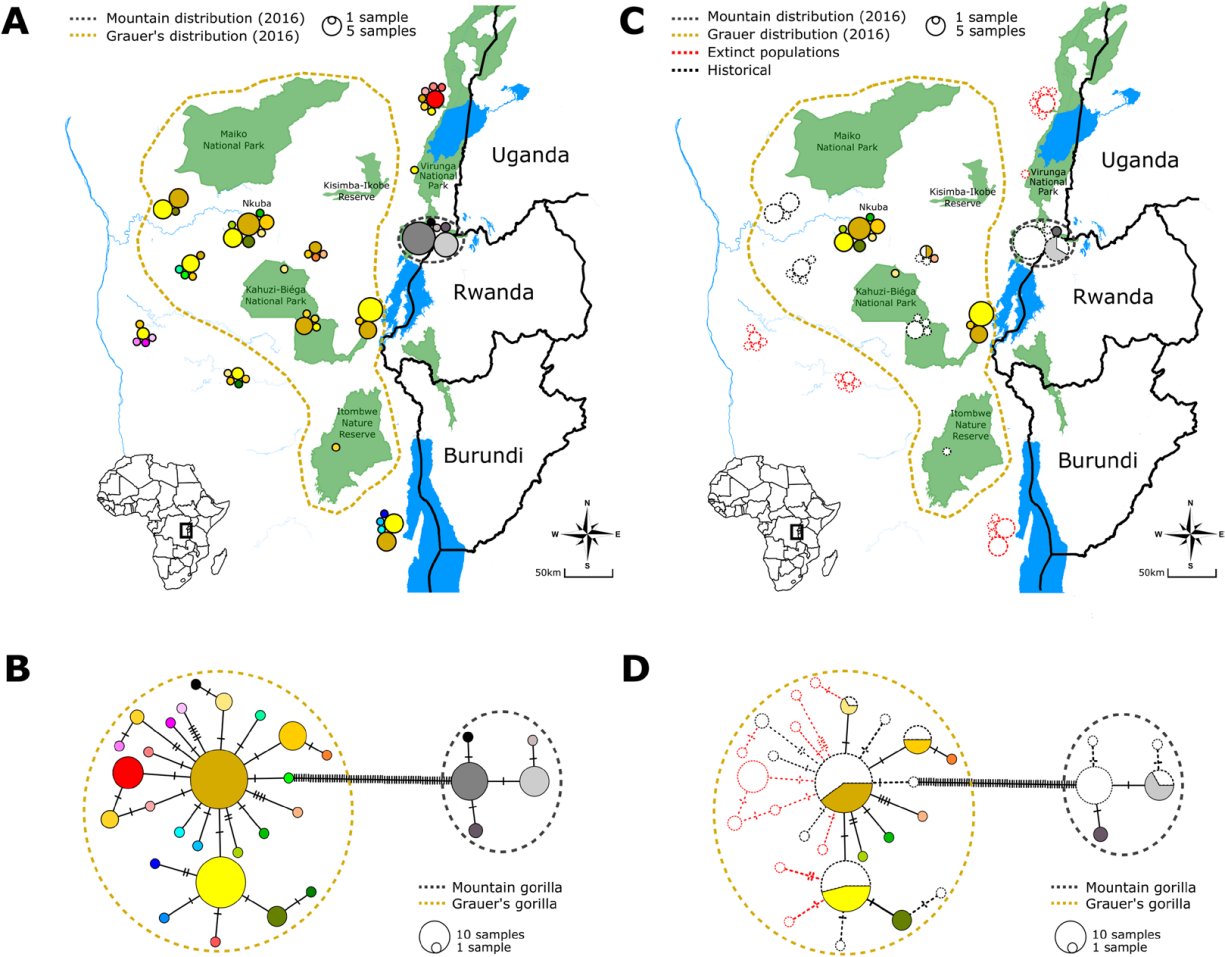
Haplotype map and haplotype network showing the geographic and genetic placement of haplotypes for both historical and modern samples. Each haplotype is marked by a unique colour. Dashed line in panels A and C designates the currently estimated distribution range of Grauer’s gorillas based on expert knowledge^26^. Distribution of studied mountain gorilla samples is limited to the Virunga Massif, but is presented larger on the map for clarity. (**A**) Geographic location of all samples and (**B**) the corresponding mtDNA haplotype network. (**C**) Geographic location and (**D**) the corresponding mtDNA haplotype network of modern samples (colored) and historical samples (shown as outlines). Red outlines designate historical samples from locations outside the current distribution range, where Grauer’s gorillas are extinct today. Maps were obtained from ^©^OpenStreetMap contributors^83^ and modified in Inkscape 0.92 (https://inkscape.org/)

Historical Grauer’s gorilla samples were obtained throughout the current species distribution range and also included areas where this species is extinct today^26,62^ (Fig. 3). Modern fecal sample collection was limited to two regions: the high-altitude sector of the KBNP on the eastern border of the species range and NK, a population from the centre of the subspecies’ range (Fig. 3). The provenance of most published Grauer’s gorilla sequences is recorded as the confiscation location, frequently a trading center^22,44^ (Table S2). Hence, in most cases, the exact geographic origin of published sequences is uncertain.

Unique haplotypes among the historical Grauer’s gorilla samples were predominantly found in the peripheral populations (Fig. 3). As result, haplotype diversity for historical samples outside of the current distribution was significantly higher than for historical and modern samples within the range (P_hist. outside vs. hist. inside_ < 0.001, P_hist. outside vs. modern inside_ < 0.001, Table S6, Fig. 2C). For historical samples, this result is unlikely the consequence of differences in sample size, as these were highly similar (n = 31 outside the current range and n = 37 within the current range, Table S6). Especially the southernmost (Baraka Sibatwa forest) and the northernmost (around Lubero and Butembo) populations, both extinct today, contained many unique haplotypes (n = 8). We note that six Grauer’s gorillas, of which only one is a female that is already beyond reproductive age, still remain in a highly isolated forest fragment on Mt. Tshiaberimu in the north-eastern part of the historical distribution range^63^. However, since this population has no means of long-term survival, we excluded the site from the current Grauer’s distribution range. Within the current distribution range, we did not observe a significant difference in nucleotide and haplotype diversity between modern and historical samples (P_Hd_ = 0.72, P_π_ = 0.76, 2-sample T-test, Fig. 2C, Table S6), despite more limited modern sampling. This pattern persisted after geographically limiting the distribution of historical samples to close proximity of modern sampling locations (Fig. S5). However, when including the nearby extinct historical populations, we again observed higher genetic diversity in historical samples (Fig. S5).

### Eastern gorilla population demography

**Figure 4:**
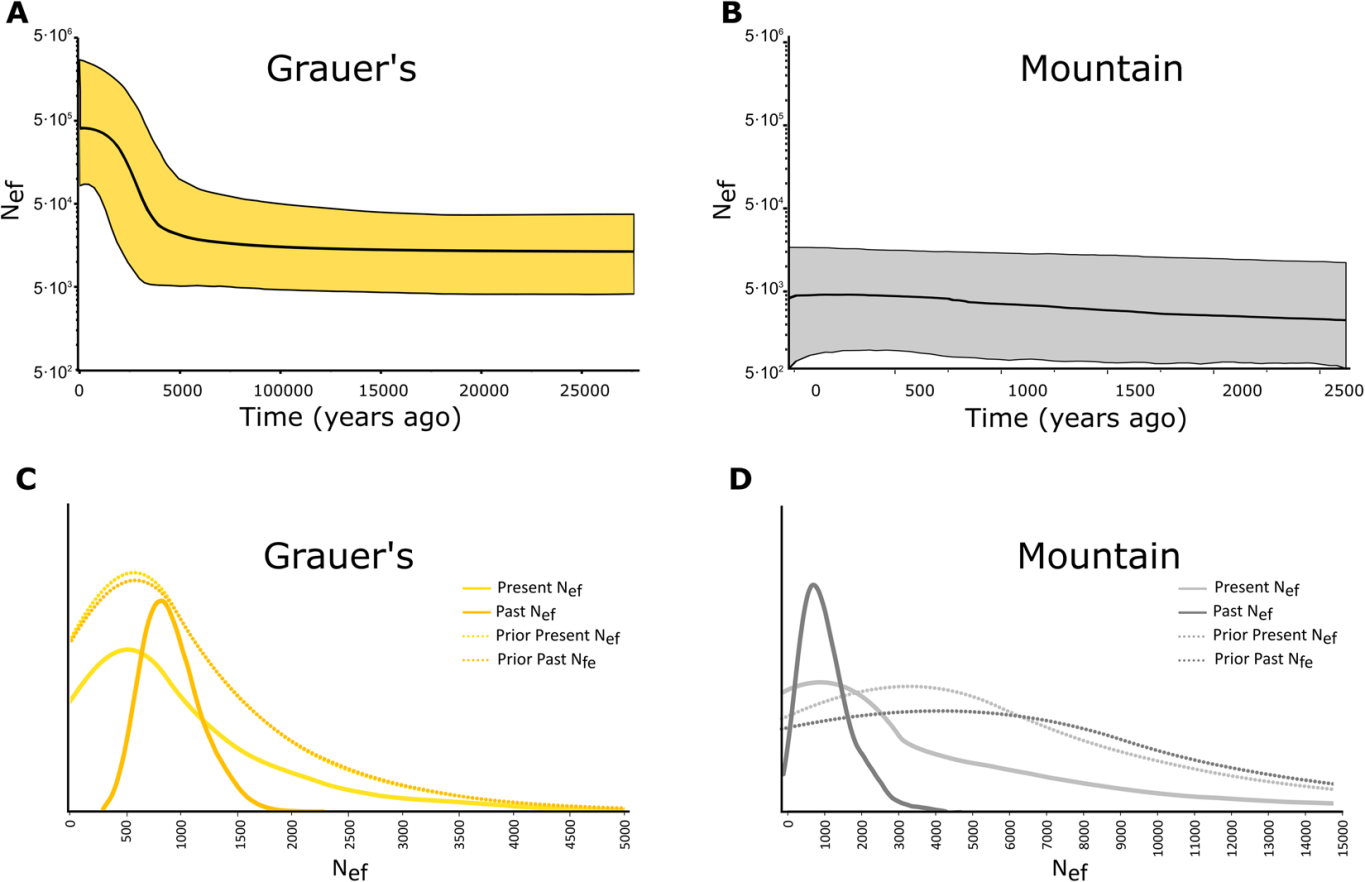
(**A**,**B**) Extended Bayesian skyline plots for both eastern gorilla subspecies. Time is presented on the x-axis (years ago, note different time scales in Grauer’s and mountain gorillas), the effective female population size, assuming a generation time of 20 years^84^, is shown on the y-axis (log-transformed). Black line shows the estimate of the mean and the colored areas corresponds to the 95% highest probability density interval. (**C,D**) Estimates of female effective population size in historical and modern Grauer’s and mountain gorillas, as inferred by approximate Bayesian computation. Probability is shown on the y-axis (unit-less). Solid lines show the inferred posterior probability distribution of present and past female effective population sizes, dotted lines depict the priors.

The significantly negative Tajima’s D (Table S4) and the star-like haplotype topology observed in Grauer’s gorillas (Fig. 1) indicate a relatively recent population expansion. This scenario is also supported by the Bayesian skyline plot analysis, which suggests a population expansion 5000 ± 2500 years ago (Fig. 4A). However, since the assumption of a single panmictic population that underlies this approach is likely violated, this result has to be interpreted with caution. In mountain gorillas, Bayesian skyline plot analysis suggested a relatively constant population size within the last 2500 years (Fig. 4B). However, this analysis is based on only four variable sites, the confidence intervals are wide and the observed pattern is therefore little informative.

Skyline plots are well known for their limitation in detecting recent or short-lasting and sudden demographic changes^64^. Therefore, to explore changes in gorilla demography within the last several decades, we employed an approximate Bayesian computation approach. We detected population size reduction in both eastern gorilla species, but the support for this model was weak, with 62% and 69% of simulations displaying a recent population decline in Grauer’s and mountain gorillas, respectively. Best-supported simulations showed on average a 4.5x (95% CI 0.3x to 16.0x) and 2.5x (95% CI 0.2x–68x) population size reduction in Grauer’s and mountain gorillas, respectively (Fig. 4C,D). However, the magnitude of reduction has to be interpreted with caution due to the wide confidence intervals (Table S7). These results are consistent with the PODs inferences, which suggested low power for inferring the direction and magnitude of population size change with our data (Fig. S6).

## Discussion

As ecosystems worldwide are facing rapid species and population loss, the importance of understanding how human activities impact natural populations has increased. However, distinguishing between recent anthropogenic and long-term demographic factors is not always trivial. Here we used a temporal approach to study recent changes in mitochondrial genetic diversity in the critically endangered eastern gorillas. By comparing temporally spaced samples, we can clearly differentiate between long-term processes that took place during the last few thousand years and short-term changes that happened in the last few decades, corresponding to only four-five gorilla generations. By including historical samples from outside of the current distribution range of Grauer’s gorillas, we were able to evaluate the importance of peripheral populations for maintenance of species-wide genetic diversity and to distinguish between signatures of population loss and general decline in species abundance.

### Historical sampling uncovers changes in genetic diversity in eastern gorillas

Our demographic modelling of the eastern gorilla populations suggested that Grauer’s gorillas experienced a period of population growth 7500 to 2500 years ago (Fig. 4A). This result is concordant with previous inferences about Grauer’s gorilla evolutionary history based on morphological analyses, and coincides with the expansion of forest habitat in central Africa^65,66^. In mountain gorillas, the extremely low levels of genetic diversity, even after including historical samples, precluded meaningful demographic inferences over the time scale of the last several thousand years (Fig. 4B).

By juxtaposing over 100 complete eastern gorilla mitochondrial genomes from modern and historical samples, we could demonstrate pronounced loss of genetic diversity in Grauer’s gorillas within the last century (Fig. 2). The use of mitochondrial markers to describe population-level processes is justified in gorillas, a species that is characterized by female dispersal^67,68^. Combined with our relatively large sample size, we therefore likely captured a reasonable representation of the Grauer’s gorilla population as a whole, even with relatively limited modern sampling.

We detected only four different haplotypes in the historical mountain gorilla population, suggesting that mitochondrial diversity was already extremely low historically. The number of unique haplotypes was limited to only two in the modern samples. Such low levels of diversity in the datasets make it impossible to derive any statistically meaningful conclusions about temporal changes in haplotype and nucleotide diversity. However, ABC modelling supports the scenario of a recent population size decrease in both eastern gorilla subspecies (Fig. 4C,D). The best supported models suggested a 25–75% reduction in effective female population size in both eastern gorilla subspecies, albeit with wide confidence intervals and low statistical power (Fig. 4, Fig. S6). This observation is also concordant with signs of very recent inbreeding in both eastern gorillas^22^. It is furthermore corroborated by known historical events that impacted Grauer’s and mountain gorillas during the last few decades, including human encroachment, civil war, and on-going political unrest in the DRC, which are associated with high rates of bushmeat hunting and deforestation^8,27,69,70^. For instance, the only closely monitored Grauer’s gorilla population of KBNP suffered an estimated 50% loss in 1999^71^. Bushmeat hunting linked to artisanal mining and the activity of numerous armed militia remains a major threat to wildlife throughout Grauer’s gorilla range^26^. As a result, Grauer’s gorilla populations continue to decline dramatically and total population loss has been estimated to be up to 90% in the last two decades^26^. In contrast, intense conservation efforts have allowed the Virunga Massif mountain gorilla population to almost double in size since the early 1980s^24^.

Present-day mitochondrial diversity in the eastern gorillas is currently the lowest of all great apes: It is over 20 times lower than in *Homo sapiens*^72^, almost 50 times lower than in eastern chimpanzee (*Pan troglodytes schweinfurthii)* and the bonobo (*Pan paniscus)*^45^, and around 100 times lower than in Sumatran orangutan (*Pongo abelli)*^73,74^. We also detected pronounced differences in mitochondrial diversity among gorilla species: western lowland gorillas harbor many highly diverse haplotypes, whereas only a few, closely related, haplotypes are found in the eastern species, particularly in mountain gorillas. These estimates are consistent with previous studies based on the mitochondrial hypervariable region^75–77^, nuclear data^22,25,44^, and match both current and historical census population size estimates^22,48^.

Our temporal approach and the use of complete mitochondrial genomes constitute a powerful strategy for detecting recent population size changes in threatened and endangered species. The extremely low levels of genetic diversity found in eastern gorillas would make sequencing of only a small part of the mitochondrial genome prohibitively uninformative (Fig. S3).

### Extirpation of peripheral populations is the main cause for the loss of genetic diversity

Grauer’s gorillas have not only experienced reduction in population size and increased fragmentation throughout their range^26,70^, but also a general range contraction, accompanied by local population extirpation^62^. Because our historical sampling contains populations from both within and outside of the current species’ distribution range, it allowed us to effectively evaluate the effect of population size reduction compared to population loss. We find that genetic diversity within the Grauer’s gorilla core distribution range remained relatively stable over the study period (Fig. 2C), despite reported decline in encounter rates^26^. However, genetic diversity in the species overall declined significantly as the result of extirpation of populations that are outside the current species range. This result holds also after accounting for differences in geographic distribution between historical and modern samples (Fig. S5). We found a particularly large number of unique mitochondrial haplotypes in these peripheral historical populations and detect multiple unique haplotypes even within relatively small geographic regions (Fig. 3). The observed pattern is possibly a result of long-term historical isolation of peripheral populations, which, by limiting (female) dispersal, may have allowed multiple unique variants to drift to high frequency. This interpretation is in line with early surveys that reported pronounced habitat fragmentation within the Grauer’s gorilla range already in the 1950s^78^.

## Conclusions

The significant loss of mitochondrial diversity in eastern gorillas over a short time period is alarming, as it likely also holds for autosomal variation (albeit to a lesser extent due to differences in effective population size), which is directly linked to the species’ evolutionary potential and hence long-term survival. Populations with low levels of standing genetic variation struggle to adapt in fast changing environments^79,80^, and it is this rapid, human-induced change that many species, including gorillas, are facing today. The recent declines in effective population size suggested by the ABC analyses may also have consequences for genetic load in eastern gorillas. Whereas genetic purging under slow inbreeding may remove deleterious variants, rapid loss of genetic diversity has been shown to have detrimental effects^15^.

Our findings stand in contrast to the common notion that small peripheral populations contain few unique genetic variants^81,82^ and instead identifies peripheral gorilla populations as important keepers of genetic diversity, highlighting their conservation importance. This observation is likely to apply to fragmented populations of many endangered species. Therefore, conservation actions aimed at preserving peripheral populations, improving habitat connectivity and hence facilitating gene flow, could have a disproportionally positive effect on maintaining overall species diversity.

## Electronic supplementary material

Supplementary figures

Supplementary tables
