# Supplementary material for: Significant loss of mitochondrial diversity within the last century due to extinction of peripheral populations in eastern gorillas
Source: Sci Rep. 2018 Apr 25;8:6551. doi: 10.1038/s41598-018-24497-7 (PMC5917027; doi:10.1038/s41598-018-24497-7)
Supplement: Supplementary file 1 — Supplementary figures [file 41598_2018_24497_MOESM1_ESM.docx]

**Supplementary materials for:**

**Significant loss of mitochondrial diversity within the last century due to extinction of peripheral populations in eastern gorillas**

*Tom van der Valk, Edson Sandoval-Castellanos, Damien Caillaud, Urbain Ngobobo, Escobar Binyinyi, Radar Nishuli, Tara Stoinski, Emmanuel Gilissen, Gontran Sonet, Patrick Semal, Daniela C. Kalthoff, Love Dalén, Katerina Guschanski*

**This PDF includes:**

Figure S1 2

Figure S2 3

Figure S3 4

Figure S4 5

Figure S5 6

Figure S6 7


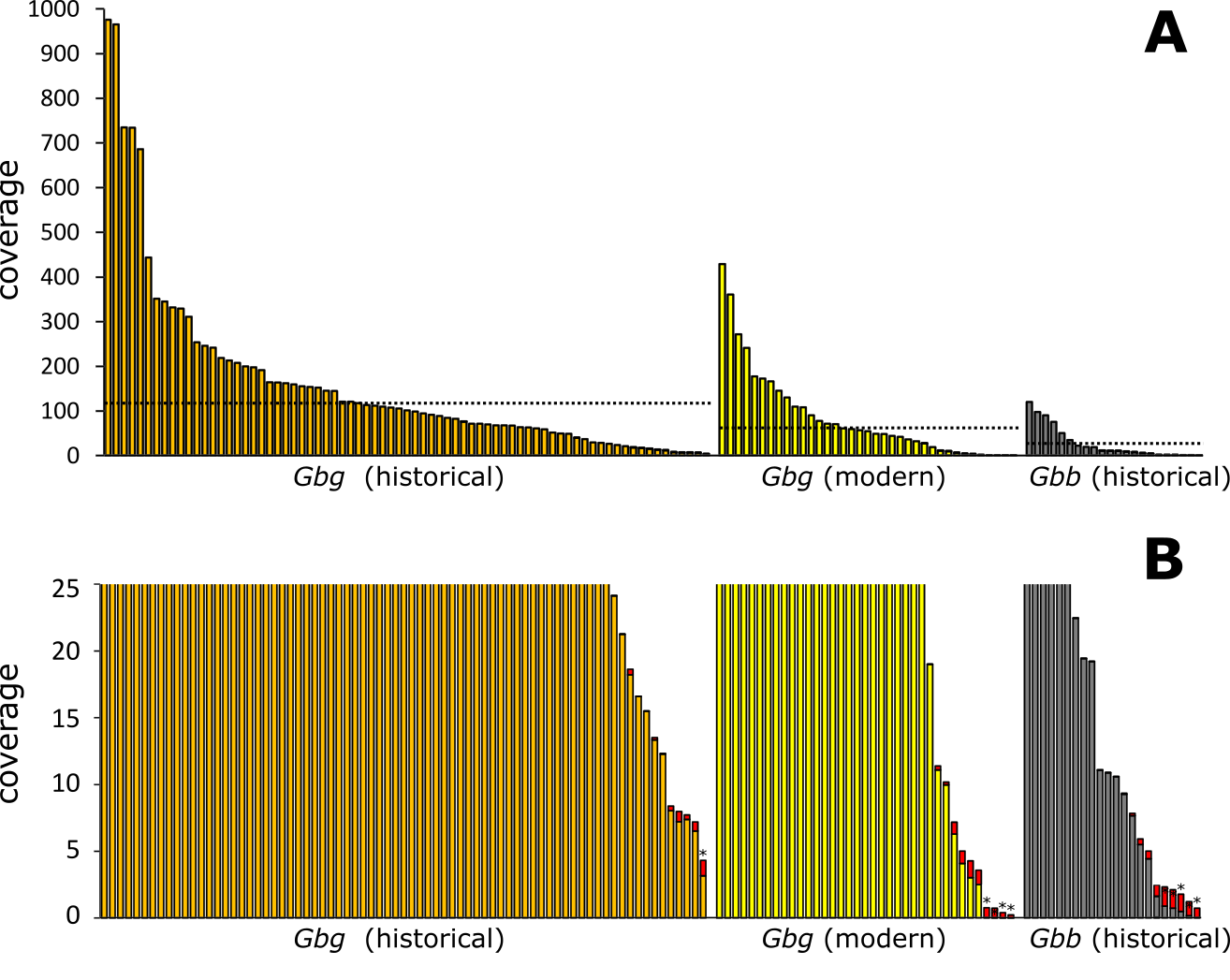


***Fig. S1****. A) Mitochondrial coverage per sample. Dotted line show averages of the three different sample types B) Same as A with different Y-axis scale. Red part of the bar shows the fraction of the mitochondrial genome covered by less than three independent sequencing reads. Asterisk shows samples removed from further analysis.*

***
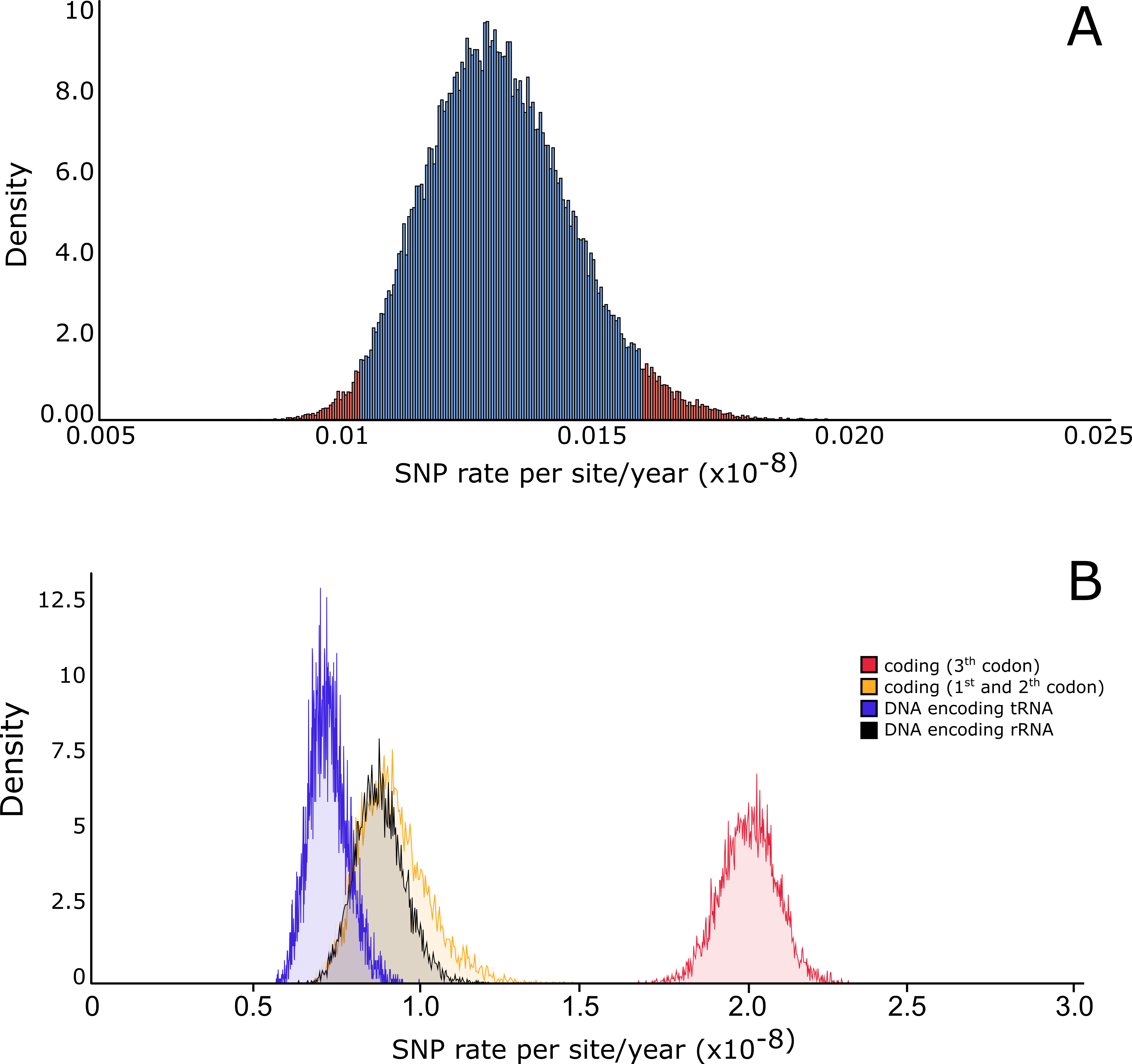
Fig. S2.*** *Gorilla-specific mitochondrial mutation rates as obtained from Bayesian modelling in BEAST. We estimated mitochondrial genome-wide mutation rate (A) which was used as input for the ABC modelling and mutation rate of the different partitions (B) which was used to construct the Skyline plots.*

***
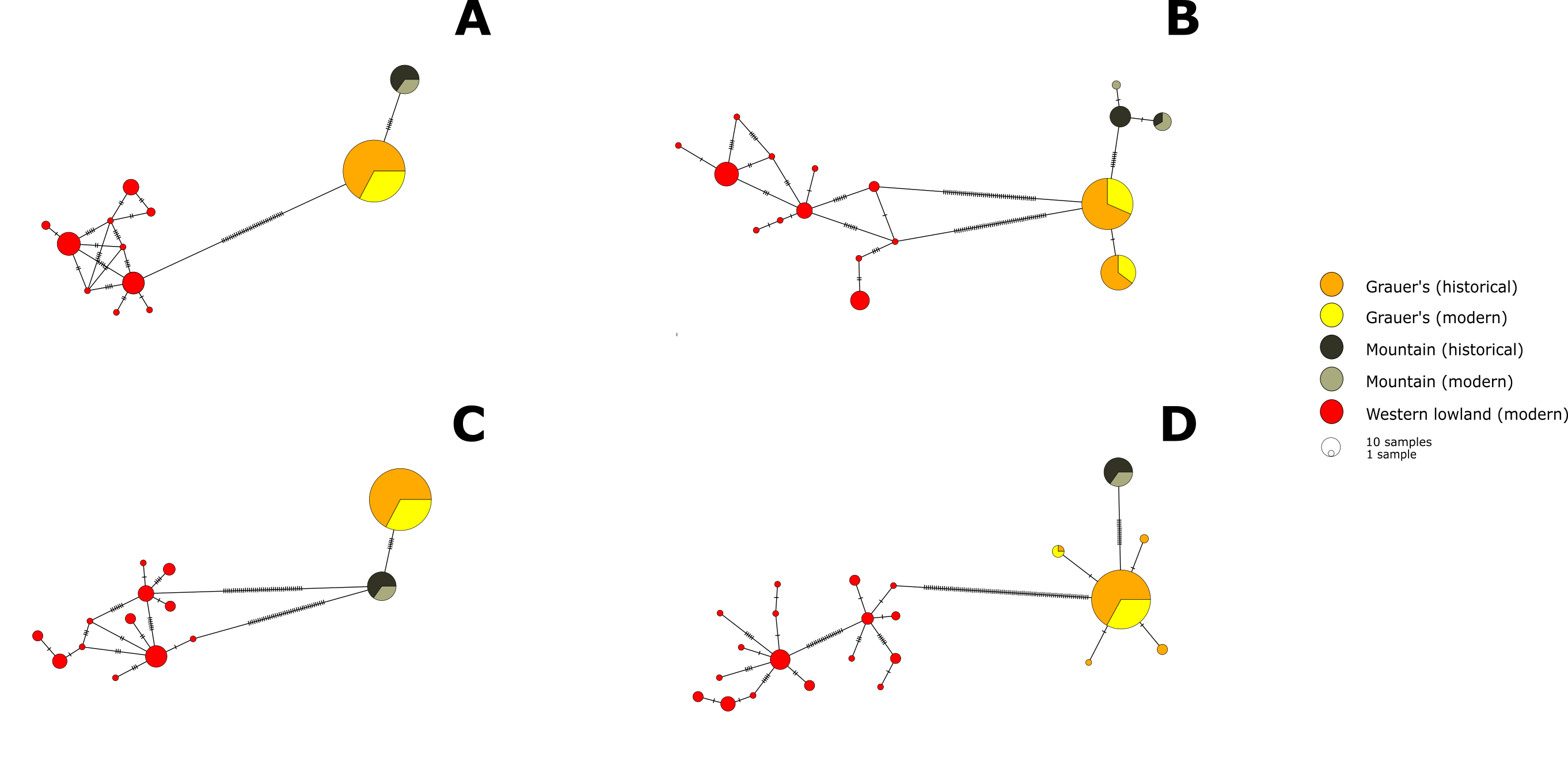
***

***Fig. S3****. Minimum spanning haplotype network for all samples based on A) 16S rRNA, B) Cytochrome b, C) Cytochrome c oxidase subunit I and D) Cytochrome c oxidase subunits 1, 2 and 3 combined.*

*
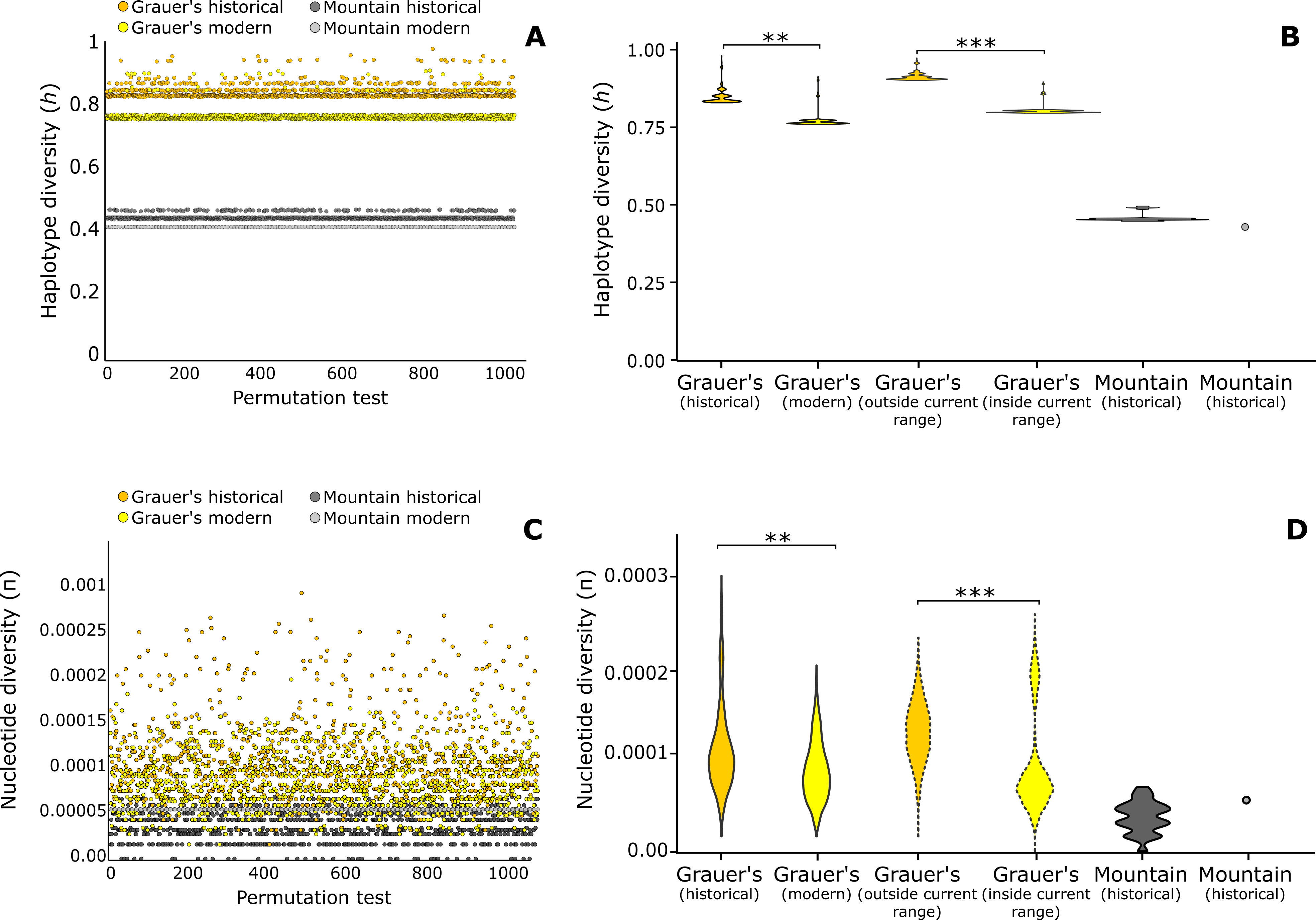
*

***Fig. S4****. Permutation test to assess the effect of sample size on genetic diversity estimates in mountain and Grauer’s gorillas. Each population was repeatedly (1000 times) randomly subsampled to eight individuals and diversity measures were calculated for each dataset. A) and B) Haplotype diversity for each population (** < 0.01, *** < 0.001). C) and D) Nucleotide diversity for each population (** < 0.01, *** < 0.001).*


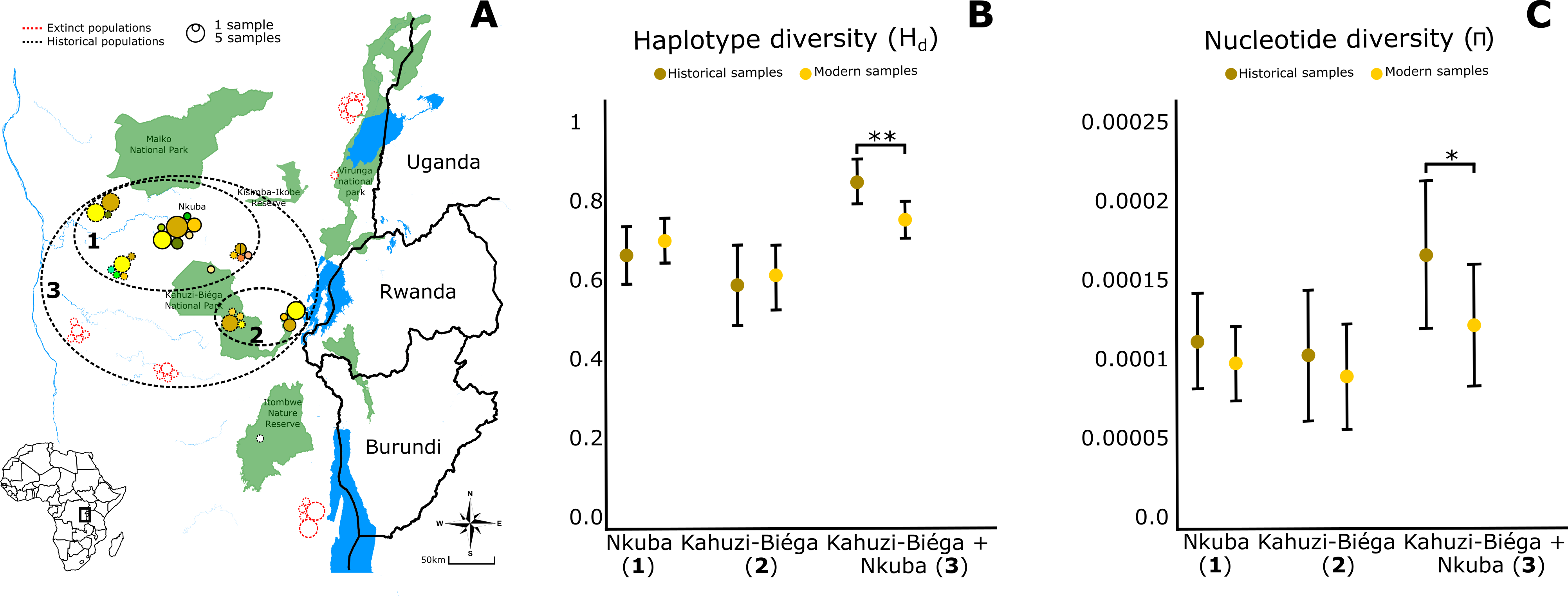


***Fig S5*** *. Local comparison of haplotype diversity for historical and modern* Grauer’s gorilla samples. A) Geographic placement for different subsampling strategies: 1) within the current Grauer’s gorilla distribution range in the proximity of the the Nkuba region; 2) within the current Grauer’s gorilla distribution range in the proximity of the high altitude sector of the Kahuzi-Biega National Park; 3) current and historical Grauer’s gorilla distribution range centered on the two modern populations (Nkuba and Kahuzi-Biega National Park). B) Haplotype diversity and C) nucleotide diversity within the geographically subsampled regions. Within the current Grauer’s gorilla distribution range no significant differences are observed between historical and modern samples from the same regions. However, haplotype and nucleotide diversity of historical samples is significantly higher as that of modern samples when nearby extinct populations are included in the comparison.*

**we excluded previously published modern mitochondrial genomes from this comparison, since the exact geographic origin of these samples is uncertain.*


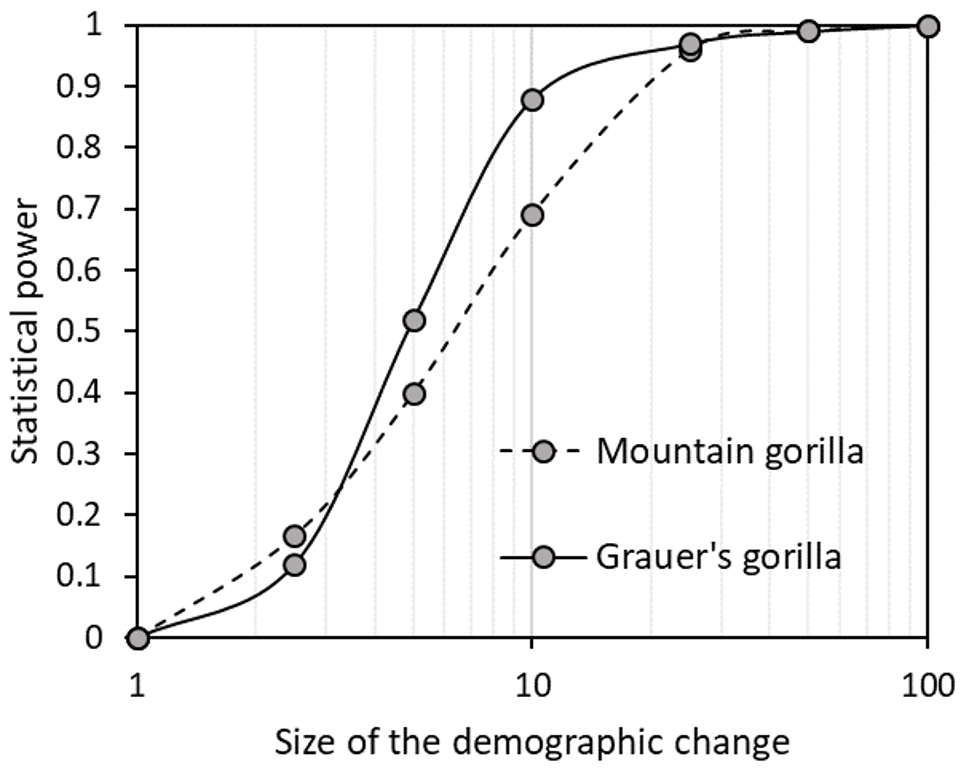


***Fig. S6****. Pseudo observed datasets (PODs) analysis showing the statistical power (on the y-axis) as function of the size of a recent demographic change in Grauer’s and mountain gorillas. Note that a demographic change of 1.0 corresponds to no-change, so the value represents the probability of rightly accepting the null hypothesis rather than the lack of statistical power to detect a demographic change.*
